# Effectiveness of coordinated care to reduce the risk of prolonged disability among patients suffering from subacute or recurrent acute low back pain in primary care: protocol of the CO.LOMB cluster-randomized, controlled study

**DOI:** 10.3389/fmed.2023.1156482

**Published:** 2023-06-20

**Authors:** Aline Ramond-Roquin, Cyril Bègue, Jonathan Vizzini, Sidonie Chhor, Tiphanie Bouchez, Elsa Parot-Schinkel, Anthéa Loiez, Audrey Petit, Maria Ghali, Matthieu Peurois, Céline Bouton

**Affiliations:** ^1^Département de Médecine Générale, Univ Angers, Angers, France; ^2^Univ Angers, Univ Rennes, EHESP, Inserm, IRSET-ESTER, Angers, France; ^3^Département de Médecine de Famille et de Médecine d’Urgence, Université de Sherbrooke, Québec, QC, Canada; ^4^Département de Médecine Générale, Univ Rennes, Rennes, France; ^5^Département d'Enseignement et de Recherche en Médecine Générale, RETINES, HEALTHY, Université Côte d'Azur, Nice, France; ^6^Biostatistics and Methodology Department, University Hospital of Angers, Angers, France; ^7^Delegation for Clinical Research and Innovation, University Hospital of Angers, Angers, France; ^8^Département de Médecine Générale, Univ Nantes, Nantes, France

**Keywords:** low back pain, subacute, acute, primary care, general practitioner, physiotherapist, coordinated care

## Abstract

**Background:**

Low back pain (LBP) is a common musculoskeletal condition and, globally, a leading cause of years lived with disability. It leads to reduced social participation, impaired quality of life, and direct and indirect costs due to work incapacity. A coordinated approach focusing on psychosocial risk factors, active reeducation, and the early use of tools to maintain employment, may be effective for improving prognosis of patients with LBP. Primary care professionals and multidisciplinary teams, who see patients in the early stages of LBP may be in the best position to implement such a coordinated approach. We designed this study to assess a coordinated multi-faceted strategy in primary care for patients with subacute or recurrent acute LBP.

**Methods:**

The CO.LOMB study was designed as a multicentric, cluster-randomized, controlled study. Patients aged 18–60 years, with subacute or recurrent acute LBP are eligible. Patients also need to be employed (but can be on sick leave) with access to occupational health services. The clusters of GPs will be randomized (1:1) to either the Coordinated-care group or the Usual-care group. Patients will be assigned the group allocated to their GP. The healthcare professionals (GPs and associated physiotherapists) allocated to the Coordinated-care group will perform a 2-session study training. The following interventions are planned in the Coordinated-care group: exploration and management of psychosocial factors, active reeducation with a physiotherapist, the implementing of tools to maintain employment, and a reinforced cooperation between primary healthcare professionals. The primary objective is to assess the benefit of coordinated primary care to reduce disability in LBP patients at 12 months after enrollment: measure using the validated French version of the Roland Morris Disability Questionnaire. Secondary objectives include the evaluation of pain, work status, and quality of life at various time points. The study plans to enroll 500 patients in 20 GP clusters. Patients will be followed up for 12months.

**Discussion:**

This study will evaluate the benefit of a coordinated multi-faceted strategy in primary care for patients with LBP. Notably whether this approach will alleviate the associated disability, attenuate pain, and promote the maintenance or return to work.

**Clinical Trial Registration:**

NCT04826757.

**Graphical Abstract:**
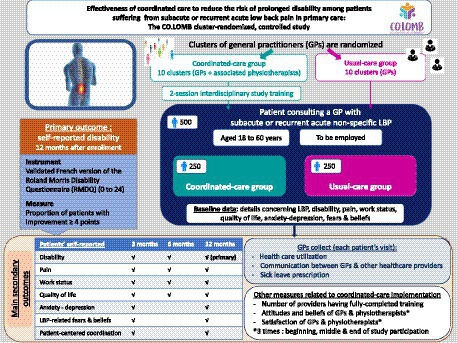


## Background

Low back pain (LBP) is a common musculoskeletal condition. Worldwide, in 2019, 568.4 million suffered from LBP, with an estimated age-standardized point prevalence of 6972.5 per 100,000 people (7.0%) (1). In Western Europe, the estimated age-standardized point prevalence was 9445.4 per 100,000 people (9.4%) (1). Even more concerning, globally in 2017, LBP was the leading cause of years lived with disability (2).

LBP can be classified according to its duration: acute (pain lasting less than 4 weeks), subacute (pain lasting between 4 and 12 weeks), and chronic (when pain has been present for more than 12 weeks). For most people, LBP improves noticeably during the first 4–6 weeks (3). After this period, LBP improves at a slower rate. At 1 year, many patients still experience low to moderate levels of pain and disability (3). Chronic or recurrent LBP is characterized by functional disability but is also accompanied by psychosocial problems, including anxiety, depression, reduced social participation, eroded family relationships, impaired quality of life, and either temporary or extended work incapacity (4–6). There are direct healthcare costs associated with LBP treatment (4, 5, 7, 8), but also, substantial indirect costs, particularly those related to prolonged work incapacity.

The traditional biomechanical approach of prescribing rest and pain medication are often ineffective (9). The biopsychosocial model for LBP emphasizes the importance of psychosocial risk factors, including psychological, psychiatric, occupational, and social factors. These factors significantly impact LBP and increase the risk for chronic LBP (10). This LBP model suggests that optimal management may be a coordinated multi-faceted strategy targeting different types of risk factors and involving various healthcare professionals.

Most patients with LBP consult general practitioners (GPs) and physiotherapists. Indeed, a French study found that 77% of patients with LBP consulted GPs and 30% underwent physiotherapy (11). Furthermore, occupational healthcare professionals in coordination with GPs play a key role in maintaining employment in these patients (12). In France, all employed workers as well as some self-employed workers have access to an occupational physician (OP) or an occupational nurse, depending on the location of their company. Comprehensive and coordinated care are critical components of primary care, with GPs playing a central role.

In this study we aim to assess the benefit of a coordinated multi-faceted primary care strategy for reducing disability, compared to usual care, in patients, aged between 18 and 60 years, with subacute or recurrent acute LBP.

## Method/design

### Study design

The CO.LOMB study was designed as a multicentric, cluster-randomized, controlled study. The clusters corresponded to at least 4 GPs practicing in the same geographic area (most often within the same multidisciplinary team). The clusters of GPs identified are located in 4 geographic regions: each attached to a University Department of General Practice (Angers, Nantes, Rennes, and Nice). The clusters will be randomized to either coordinated primary healthcare (the Coordinated-care group) or usual care (the Usual-care group).

### Study population

All patients, presenting with LBP at the practices of GPs participating in the study, will be considered for study participation. The eligibility criteria for the study are as follows:

#### Inclusion criteria

Patients need to meet the following criteria to participate in the study:

1. Patients aged between 18 and 60 years old.
2. Patients consulting a GP with LBP, defined as a pain situated between the 12th rib and the gluteal cleft. The LBP must be either:

- Subacute LBP, defined as back pain lasting between 4 and 12 weeks and preceded by at least 30 days without back pain.
- Acute recurrent LBP, defined as back pain lasting for less than 4 weeks and preceded by at least 30 days without back pain. The patients must have consulted a healthcare provider for LBP within the previous 12 months.

3. Patients must be employed (but can be on temporary sick leave) at enrollment.
4. Patients must have access to occupational health services (working for a company that either has its own OP or a company using shared occupational health services).
5. Patients must provide signed consent to participate in the study.
6. Patients must be registered with a social security scheme.

#### Non-inclusion criteria

Patients meeting any of the following criteria will not be eligible for the study:

1. Patients with a specific LBP, including LBP due to fractures, infections, osteoporosis, inflammatory diseases, or tumors (with confirmed diagnosis or highly suspected, resulting in specific and/or urgent treatment).
2. Patients with LBP with pain irradiating below the knee.
3. Patients for whom active reeducation is contraindicated.
4. Patients performing follow-up for their LBP with a GP not participating in the study.
5. Patients performing reeducation with a physiotherapist not participating in the study and who are unwilling to change physiotherapist (Interventional group only).
6. Patients planning to leave the study territory within the 12 months following study enrollment.
7. Patient planning to retire within the 12 months following study enrollment.
8. Pregnant, breastfeeding, or parturient women.
9. Patients undergoing psychiatric care under duress.
10. Patients admitted into a social or healthcare center for a reason other than for research.
11. Patients unable to read and write in French.
12. Persons deprived of their liberty by judicial or administrative decision.
13. Adult patients under legal protection measure (guardianship).
14. Persons unable to provide consent.

### Randomization

GPs will be cluster randomized for the study. The clusters of GPs will be randomized, in a 1:1 ratio, to either the Coordinated-care or the Usual-care group. The randomization will be stratified by the geographical region related to the University Department of General Practice (Angers, Nantes, Rennes, and Nice). The study plans to enroll 20 GP clusters distributed as follows: 10 clusters for the Department of General Medicine attached to Angers (5:5), 4 for each of those attached to Nantes (2:2) and Rennes (2:2), and 2 for that attached to Nice (1:1). The randomization of the clusters for the Department of General Medicine attached to Angers (10 clusters) will also be stratified by the time of study initiation, since the synchronous initiation of the clusters in Angers is not feasible. The randomization will be performed by the Biostatistical Department of the University Hospital Center (“CHU”) at Angers. All patients followed up in the same cluster will be allocated the same group as their GP.

### Interventions

Prior to the study, all healthcare professionals (GPs and associated physiotherapists) in the clusters allocated to the Coordinated-care group will undergo a 2-session training for the study interventions. The training sessions will be performed within each of the 10 clusters. Each training will be comprised of two sessions separated by a 2-to-4-week time interval. The first 90-min training session will focus on three blocks: the factors that may influence the evolution and treatment of LBP, the French recommendations that promote active LBP management with therapeutic education (13), and the tools available for maintaining employment in patients with LBP. This first session will be performed autonomously by GPs and physiotherapists, guided by a video. The professionals will be asked to produce a written summary of their exchanges on each block. During the 2–4-week interval between the training sessions, the GPs and physiotherapists will be invited to complete auto-observation questionnaires concerning any patients with LBP that consult them. The second session will consist of a three-and-half-hour in-person training, at each cluster’s location, by the clinical training team: made up of the same doctor and physiotherapist. In addition, the clinical training team will systematically invite a local OP to this second session. The session will combine formal presentations related to each interventional component (psychosocial factors in LBP, active exercise reeducation program for patients with LBP, and the tools to maintain employment). The training session will also include several periods of time for the health care professionals to exchange their clinical experiences, and to propose ways to evolve their practices to incorporate the elements associated with the study intervention. During the session there will be a final sequence focusing on interprofessional collaboration, during which the healthcare professionals will decide on the modalities for collaboration to be implemented in the study.

The aim of the training will be for the healthcare professional to appropriate the concepts, tools, and interventions applied during the study. During the training, healthcare professionals will also discuss various aspects of healthcare including care providers, resources offered in the region, as well as potential barriers to collaboration, and how to overcome these barriers.

Healthcare professionals in clusters allocated Usual-care received no study-specific clinical training.

The primary care interventional components in the Coordinated-care group will be as follows:

- Exploration and management of psychosocial factors

Healthcare professionals (GPs and physiotherapists) will be asked to explore, during their consultations, various psychosocial factors, including individual psychological, psychiatric, cognitive-behavioral, family factors (usually named as “yellow flags”), socio-economic factors (usually named as “blue flags”), and socio-occupational factors (usually named as “black flags”). The factors impacting the transition from acute to chronic LBP and the appropriate clinical care will be discussed in detail during the training (4, 10, 14, 15). Furthermore, eligible patients, allocated to the Coordinated-care group will be systematic given the French social security brochure, “I suffer from LBP: what is it and what should I do?” (translated from the French: “Je souffre de lombalgie: de quoi s’agit-il et que faire?”) by their GP (16). The brochure will help to educate the patient concerning the evolution of LBP and to eliminate false beliefs.

During the study, the GP will continue to follow the patients according to the patients’ individual needs and preferences, as assessed by the patients and their GP. The general recommendation will be to perform regular follow-up visits (for example weekly or every 2 weeks, at least during the early phase of medical care, and especially in the case of sick leave) until LBP-related complaints have been resolved. No specific frequency for the consultations will be imposed by the protocol since the overmedicalization of LBP is known to promote the development of chronic LBP (17). In particular, the duration of disability is known to increase with the number of healthcare consultations, with referrals to specialists, and performing early diagnostic imagery (18).

- Active exercise reeducation program

Patients in the Coordinated-care group will have access to individual reeducation by a physiotherapist trained for the study. The reeducation will be composed of an intensive exercise rehabilitation program. The program comprises of up to 15 sessions of 1 h, 2–3 times per week and included therapeutic education. This approach is commonly recommended for treating LBP (19), but not frequently implemented (due to limited availability of physiotherapists and/or costs for patients). In clinical trials evaluating the efficacy of these programs, these programs usually last between 8 and 31 h. Depending on the physiotherapist’s assessments during the program and the patient’s needs and preferences, the program can be stopped before the 15^th^ session or can be extended with maintenance therapy sessions.

- Use of tools to maintain employment

GPs and physiotherapists in the Coordinated-care group will be trained during the study to use the tools to maintain employment and will be encouraged to implement these tools early during LBP management to prevent prolonged incapacity at work, extended sick leave or even job loss.

GPs and physiotherapists will be asked to systematically inquire about the occupational situation of their patients throughout the study to better appreciate the evolution of the patient’s situation and to adapt the clinical strategy.

The following recommendations and tools, for maintaining employment will be proposed:

✓ GPs and physiotherapists will be requested to systematically refer their patients to either their OP or an occupational nurse (depending on the healthcare organization and the resources available) within 15 days after enrollment.
✓ GPs and physiotherapists will be asked to encourage their patients to return to work as early as possible, considering their clinical and occupational situation. Also, health professionals should favor short periods of sick leave, of 1–2 weeks (particularly during the early stages of LBP), instead of long periods. Also, patients will be systematically proposed an appointment with their GP before returning to work.
✓ GPs and physiotherapists will be requested to use the tools to help maintain employment available in France in all relevant situations (12). During the study training, GPs will be trained to use the 3 main tools: a visit with the OP before returning to work after a sick leave (referred to as the “pre-return-to-work visit”), a progressive return to work based on part-time work for therapeutic reasons (referred to as “therapeutic part-time work”), and the “recognition of handicapped worker status.” These tools will be discussed in detail, including their usefulness, their limitations, and how to implement them in practice, considering local resources and the healthcare organization.

- Increased cooperation among healthcare professionals for a coordinated care for patients

The cooperation between healthcare professionals within a cluster, allocated the study interventions, will be initiated during the study training as an explicit component of the intervention. The cooperation will be facilitated by the proximity of healthcare professionals in the study. At the portion of the training session dedicated to the collaboration between healthcare professionals, time will be allocated for the professionals to discuss obstacles, opportunities for and modalities to collaborate. The use of tools available locally, but underused, including shared information systems and multidisciplinary meetings, will be encouraged for patients included in the study. The GPs will be instructed, with permission from the patient, to provide the physiotherapists not only with the prescription but also the relevant clinical information at the start of treatment. The physiotherapists will be instructed to provide the GP with a final report including an assessment of the patient’s condition once the patient completed the reeducation program. Also, when justified by the patient’s clinical situation, communication between GPs and physiotherapists will be encouraged throughout the reeducation program. Furthermore, the healthcare professionals will be encouraged to correspond or initiate communication with all healthcare professional implicated in the patient’s treatment and return to work, even those not participating in the study. Finally, the following 3 templates for letters will be provided by the research team: a reference letter template from the GP to the physiotherapist, a final report template from the physiotherapist to the GP at the end of reeducation program, and a template from the GP to the OP or occupational nurse. Professionals in the clusters allocated to the Coordinated-care group will be instructed to locally adapt these templates as required and/or to use their own templates.

This multi-faceted study intervention was designed as a coordinated and comprehensive biopsychosocial healthcare strategy. The intervention is fundamentally patient centered: adapted to the needs and preferences of each patient. The healthcare professionals must evaluate the pertinence of each part of intervention according to the patient’s specific situation, and to incorporate the patient’s preferences in the treatment decision, following a shared decision approach. The patient will remain free to accept or not, or to delay, any proposed part of intervention without being excluded from the study.

Patients in clusters allocated usual care are treated according to the GP’s usual practice.

To avoid creating a feeling of injustice in healthcare professionals allocated the non-intervention group and to motivate them to actively participate in the study, the 2-session training will be offered to physicians and physiotherapists of these clusters after the end of the study.

### Primary objective and outcome

The primary objective is to assess the benefit of coordinated care for reducing disability, compared to usual care, in patients, aged between 18 and 60 years, consulting for subacute or recurrent acute LBP. The primary objective will be measured using the validated French version of the Roland Morris Disability Questionnaire (RMDQ) (20–22). The questionnaire comprises 24 questions and is scored from 0 (without disability) to 24 (with maximum disability). The primary outcome measure is the proportion of patients that has an improvement (lower score) of at least 4 points at 12 months after enrollment.

### Secondary objectives and outcomes

The benefit of coordinated primary care, in terms of patients’ clinical improvement and employment status will also be assessed using the following secondary outcome measures:

1. The proportion of patients that have improved RMDQ scores by at least 4 points, relative to baseline, at 3 and 6 months after enrollment.
2. The evolution of the RMDQ scores measured at baseline and then at 3, 6, and 12 months after enrollment.
3. The proportion of patients that improved by at least 2 points on the numerical pain scale, relative to baseline, at 3, 6, and 12 months after enrollment. The numerical pain scale is widely used and validated by the French Health Authority (“Haute Autorité de Santé”) (23). The scale is assessed from 0 (no pain) to 10 (maximum pain). In the literature an improvement of 1.5 points is considered to the minimum improvement to be of clinical significance (24).
4. The evolution of the numerical pain scale scores measured at baseline and then at 3, 6, and 12 months after enrollment.
5. The proportion of patients that are “actively employed,” defined as being employed and being at work (not on sick leave), at 3, 6, and 12 months after enrollment. Patients on sick leave will not be considered as being actively employed.
6. The number of days of sick leave during the 12 months after enrollment.
7. The proportion of patients considered as having “improved overall.” Patients will be considered to have “improved overall” if they have improved their RMDQ scores by at least 4 points, improved their numerical pain scale by at least 2 points, and are actively employed. The outcome will be measured at 3, 6, and 12 months after enrollment.
8. The change in the physical and mental quality of life of patients during the study measured using the Short-form 12 (SF-12). The SF-12 consists of physical and mental dimensions. Each dimension has 4 categories measure between 0 and a maximum value of 100. The higher the score the better the quality of life. The SF-12 is extensively used with a validated French version (25, 26). The SF-12 will be measured at baseline and at 3, 6, and 12 months after enrollment.
9. The changes in the anxiety and depression scores of the Hospital Anxiety and Depression Scale (HAS). The HAS comprises 14 items: 7 for anxiety and 7 for depression (27). Each item is scored from 0 to 3. The HAS provides anxiety and depression scores ranging from 0 to 21. The study will use the validated French version and will be completed by patients at baseline and at 3 and 12 months (28).

The benefit of coordinated primary care, in terms of the beliefs, feelings, and satisfaction of patients and healthcare professionals will also be assessed using the following secondary outcome measures:

1. The changes in the occupational and physical scores of the Fear Avoidance Beliefs Questionnaire (FABQ). The self-administered FABQ evaluates the patient’s fears and beliefs surrounding LBP (29). The validated French version of the FABQ was used during the study (30). The FABQ comprises 16 items divided into two dimensions: the physical (items 1–6) and the occupational (items 7–16). Each item is scored from “0” (completely disagree with the statement) to “6” (completely agree with the statement). Thus, the maximum score is 36 for the physical dimension and 60 for the occupational dimension. The FABQ will be completed by patients at baseline, and at 3 and 12 months.
2. The change in the Patient-Centered Coordination by a Care Team (PCCCT) questionnaire. The PCCCT instrument measures the quality of primary care from the patient’s perspective. The questionnaire is composed of 14 items each scored from 0 to 3 (31). The overall score will range from 0 (worst coordination) to 42 (best coordination). The PCCCT questionnaire will be completed by patients at baseline and at 3 and 12 months.
3. The change in the GPs’ satisfaction with the healthcare provided for their patients’ LBP.
4. The change in the physiotherapists’ satisfaction (only in the Coordinated-care group) with the healthcare provided for their patients’ LBP.

The GPs’ and physiotherapists’ satisfaction will be measured, on a scale from 0 (not satisfied) to 10 (completely satisfied), at the following timepoints: when the cluster is initiated, at 6 months after the 5th patient is included in each cluster, and at the end of the follow up of the last patient in each cluster. The GPs’ satisfaction will be compared between the study groups.

The level of implementation of coordinated primary care (the study intervention) will be assessed using the following secondary outcome measures:

1. The number of healthcare professionals, in the interventional group, that performed both training sessions.
2. The change in the attitudes and beliefs of physiotherapists (only in the Coordinated-care group) toward LBP. This will be measured using the Pain Attitude and Belief Score (PABS) (32). This instrument assesses treatment orientations (either biomechanical or biopsychosocial). The PABS comprises 36 items: 10 in the biomechanical and 9 in the biopsychosocial dimension. Each item is score using a 6-point Likert scale: 1 (disagree) to 6 (totally agree). This data will be collected when the cluster is initiated, at 6 months after the 5th patient is included in each cluster, and at the end of the follow up of the last patient in each cluster.
3. The change in the attitudes and beliefs of GPs (in both study groups) toward LBP. This will be measured using the biomechanical and biopsychosocial dimensions of the PABS. The GPs will complete the instrument when the cluster is initiated, at 6 months after the 5th patient is included in each cluster, and at the end of the follow up of the last patient in each cluster.
4. The numbers and modes of communication (letters, emails, facsimiles, and telephone calls), in both study groups, between GPs and other healthcare professionals (whether or not they are participating in the study) implicated in the patients’ management.
5. Number of consultations/visits/examinations for patients, in both study groups, according to the type of healthcare professional (GPs, physiotherapists, OP or occupational nurse, rheumatologists, other medical specialists, other paramedical healthcare professionals, osteopaths, emergency room visits, imagery, and other examinations), whether or not the healthcare professionals are participating in the study.

### Data collection

The schedule for collecting patient data is shown in Table 1 and that for collecting healthcare professional data in Table 2.

**Table 1:** Schedule for collecting patient data.

Study procedures	Study time points			
	Baseline	3 months*	6 months*	12 months*
Delay allowed (days)		−7 to +21	−7 to +21	−7 to +21
**Baseline procedures**				
Verification of eligibility	X			
Providing study information and obtaining signed informed consent	X			
**Collection of patient data**				
Sociodemographic data	X				Medical history (LBP and concomitant conditions)	X			
Details concerning LBP	X	X	X	X
Work and employment data (including sick leave)	X	X	X	X
**Completion of instruments by patients**				
Roland Morris Disability Questionnaire (RMDQ)	X	X	X	X
Numerical pain scale (scored from 0 to 10)	X	X	X	X
Short-form 12 (measure of the physical and mental quality of life)	X	X	X	X
Fear Avoidance Belief Questionnaire (FABQ)	X	X		X
Hospital Anxiety and Depression Scale (HADS)	X	X		X
Patient-Centered Coordination of Care Team (PCCCT) questionnaire	X	X		X
Nordic musculoskeletal questionnaire	X			X

LBP, low back pain.

*A medical visit will not be required for these study time points, since patients’ data will be collected using self-administered questionnaires completed at home.

**Table 2:** Schedule for collecting healthcare professional data.

Study procedures	Study time points		
	At cluster initiation	At 6 months after the 5th patients is enrolled in the cluster	At the end of follow up of the last patient in the cluster
**Baseline procedures**			
Sociodemographic and details concerning their healthcare practices will be collected from all GPs and only physiotherapists in the Coordinated-care group	X		
The number of healthcare professionals that underwent study training (Coordinated-care group only)	X		
**Completion of instruments by general practitioners (GPs)**			
GPs’ satisfaction with healthcare provided for their patients’ LBP (score from 0 to10)	X	X	X
Pain Attitude and Belief Score (PABS)	X	X	X
**Completion of instruments by physiotherapists (coordinated care group only)**			
Physiotherapists’ satisfaction with healthcare provided for their patients’ LBP (score from 0 to10)	X	X	X
Pain Attitude and Belief Score (PABS)	X	X	X

LBP: low back pain.

At baseline, all data will be collected using a paper version of the case report form. After baseline, all participants (patients, GPs, and physiotherapists) will collect data either via the internet (electronic case report form) or using a paper version, at their discretion.

#### Patients’ data

The baseline visit for patients is the only study-specific visit required by the protocol (Table 1). After the enrollment of patients during GP consultations, patients’ data will be collected using self-administered questionnaires completed at home. The patients will be invited by the study coordination team to complete the questionnaires during a period from 7 days before to 21 days after each evaluation endpoint. Reminders will be sent during this period in cases of non-completion. The patients will complete the standardized instruments, as well as questions concerning their LBP management and employment (including their employment status, sick leave, and/or the assistance provide to maintain active employment) since the last evaluation performed. Patients will provide data at 3, 6, and 12 months after enrollment.

During the study, data will also be collected from GPs whenever a participant consults them during the planned 12 months of follow up. The following data will be collected:

- The number of consultations/visits/examinations performed by healthcare professionals (including other GPs, physiotherapists, and OP), since the previous evaluation.
- The number and modes of communication between GPs and other healthcare professionals since the previous evaluation, including which healthcare professional initiated the exchange.
- Employment data, including details concerning sick leave.

Finally, at 12 months after enrollment of each patient, the data collected during follow up will be updated so that all information required for analyses have been provided.

#### Professionals’ data

At initiation of the clusters, sociodemographic and healthcare practice data will be collected from all GPs and only physiotherapists in the interventional group (Table 2). Furthermore, all GPs and only physiotherapists in the interventional group will assess their satisfaction with the healthcare provided for their patients’ LBP and complete the PABS at initiation of their cluster, at 6 months after the 5^th^ patient is included in their cluster, and at the end of the follow up of the last patient in their cluster.

### Sample size

To calculate the sample size required for the study we hypothesize that 50% of patients in the control group (Usual-care group) and 70% of those in the interventional group (Coordinated-care group) will have a significantly reduced disability at 12 months after being enrolled (3, 33). Considering a cluster randomized study with the following statistical characteristics:

- A power of 80%.
- A type 1 error (α-risk) of 0.05.
- A mean cluster size of 25 patients.
- A coefficient of variation in the cluster size of 0.2.
- An intraclass correlation coefficient of 0.03.
- An attrition rate of 20%.

To show a difference of 20% between the study groups (70% vs. 50%) the study needs to include 10 clusters of 25 patients in each study group. Therefore, a total of 20 clusters of 25 patients: 500 patients are required for the study. The sample size was calculated using Stata (Stata 13.1 software, package clustersamplsi).

This sample size is realistic considering that GPs on average perform 2,500 consultations per year in patients aged between 18 and 60 years (34). Among these 1 of every 200 consultations concerns subacute LBP (35). Therefore, on average 12–13 patients per year with subacute LBP consult each GP in France. The number of consultations for recurrent acute LBP is more difficult to estimate but is about as frequent as those for subacute LBP. The planned patient enrollment period is 24 months for any given cluster. Each cluster will consist of at least 4 GPs. Therefore, the study target of recruiting 25 patients per cluster is feasible. The total planned enrollment period is 36 months, comprising an initial 12-month period during which the clusters will be initiated followed by a 24-month patient enrollment period.

The “Lasagna law,” suggests that previsions of recruitment are generally optimistic for various reasons, including but not limiting to inclusion criteria (36). Therefore, in our study, to anticipate the risk of lower than expected and/or differential recruitment levels between the control and interventional groups, we developed various strategies to support patient recruitment. These include communication strategies, that were adapted according to the study group and according to each GPs recruitment activity. Moreover, the recruitment status and other study information will be communicated to GPs via newsletters and an online platform. The study team will also maintain contact will all GPs throughout the study to ensure that they remain motivated and that they recruit patients.

The coefficient of variation in the cluster size was set at 0.02. This corresponds with a mean cluster size of 25 patients, and an interquartile range of 20–30 patients. The intraclass correlation coefficient of 0.03 is conservative. Indeed, the median intraclass correlation coefficient considering the various outcome measures, including disability, have been estimated in primary care to be 0.01, with an interquartile range of 0–0.032 (37).

If patients are lost to follow-up, the GP will make all attempts to contact the patients and ensure follow-up. Patients lost to follow-up will not be replaced.

### Statistics

Quantitative data will be presented as means with standard deviations and will be compared using Mann–Whitney tests. Qualitative data will be presented as numbers with percentages and compared using Fisher’s exact tests.

For the primary objective, the proportion of patients that have an improved (lower score) of at least 4 points, in the RMDQ, at 12 months after enrollment will be compared in the study groups. The analysis will be performed using a multilevel logistic mixed model that will allow the clustering effect to be considered as random.

Similarly, the secondary outcome measures with binary outcomes compared between the study groups (e.g., the proportion of patients that improved by at least 2 points on the numerical pain scale at 12 months) will be analyzed by multilevel logistic mixed regression models.

Secondary outcome measures assessing changes in a quantitative parameter over time (e.g., the evolution of the RMDQ scores measured at baseline and then at 3, 6, and 12 months after enrollment) will be analyzed using linear multilevel mixed regression models. The individual effect will be included in the cluster effect. Time will be considered as qualitative variables with a varying number of modalities, e.g., 4 modalities for outcomes assessed at baseline, and then at 3, 6, and 12 months, and 3 modalities for outcomes assessed at baseline, and then at 3 and 6 months. The effect of the intervention will be evaluated by including an interaction between the variables for time and for the study group. The effect of the intervention will be assessed at each time point. The increased alpha-risk, due to multiple analyses, will be accounted for using the Bonferroni correction.

For these various multilevel models, the variance–covariance matrices will be considered as unstructured. Missing data will be treated using a set of 10 multiple imputations based on chained equations (38, 39). The analyses will be performed bilaterally with the alpha-risk set at 5%, except in cases where the Bonferroni correction was used. The data will be analyzed on an intent-to-treat basis. Thus, all patients with data will be included in the analysis unless they specifically indicate that they do not want their data analyzed.

## Discussion

Our study assesses a multi-faceted coordinated patient-centered strategy for treating LBP that incorporates the 4 main factors identified in the literature:

1. The management of psychosocial risk factors (10, 14).
2. Active exercise reeducation program (40, 41).
3. Tools for maintaining employment (12).
4. Reinforced cooperation between primary healthcare professionals.

These factors are supposed to be clinically relevant for reducing the risk of persistent incapacity and extended sick leave and/or job loss in people presenting with LBP. At the chronic LBP stage, the benefits of strategies based on these factors remain modest and are often short-lived. The literature suggests that implementing these strategies at an earlier stage, when LBP is subacute or acute recurrent, may be more effective in reducing the risk of chronic incapacity. Thus, strategies need to be implemented in primary care (and not in the hospital setting) where early symptoms of LBP are managed. Furthermore, primary healthcare workers have the required competence to implement these strategies. Although, at present, there is not sufficient data to confirm this hypothesis. The French (13) and International recommendations (42–44) for treating patients with a risk of chronic LBP incorporate the 4 factors mentioned above. However, these recommendations are often based on expert consensus and not on evidence-based research. To date, the results of implementing strategies in primary care, only based on certain factors, have proved to be disappointing (45–48). We hypothesize that only the simultaneous implementation of strategies based on these 4 factors will significantly reduce the risk of chronic incapacity considered to be clinically relevant.

We decided to limit our study eligibility to adults younger than 60 years old. Indeed, the etiology of LBP varies with age. In adults younger than 60, the cause of LBP is mostly general and unspecific, becoming more specific with age. Beyond 70, the specific origin of LBP becomes clinically significant (33). Moreover, a critical portion of our study concerns the occupational impact of LBP. This impact becomes more difficult and/or less relevant to assess in patients older than 60 years because of the high probability of them being unemployed or retired.

GPs in the interventional group may preferentially include patients with severe LBP instead of enrolling all eligible patients if they consider the intervention to be better than usual care, and that this improved care may benefit patients with severe LBP more than patients with less severe LBP. This phenomenon may also occur in the control group, where GPs may be reluctant to suggest study participation because of the protocol’s constraints, to patients with non-severe LBP. We have examined this issue and will address this by providing continuous support for GPs during the study. Where a significant difference between groups in terms of patient’s LBP status at baseline is observed, we planned to adjust analyses to limit the impact of this bias on the results.

Most studies that have assessed the clinical evolution of LBP have used either pain, incapacity, and/or returning to work to measure the effectiveness of interventions (33, 49). However, from a patient’s perspective, reduced LBP is mainly comprised of three dimensions: attenuated pain, improved functional capacity, and combined with an acceptable quality of life, which includes the capacity to work (24). In our study, we chose perceived disability, considered by patients to be of utmost importance, as our primary outcome measure. However, we have included several secondary outcome measures, including measures to assess pain, quality of life, and the patients’ occupational status—other important facets of LBP.

Currently, in France, primary healthcare professionals, working in the same geographical region, are encouraged to group together in multidisciplinary practices. Our study is consistent with this evolution of primary healthcare, evaluating a coordinated strategy composed of a multidisciplinary team. Moreover, our study allows healthcare professionals in each region to form networks that hopefully will persist after the study has been completed. Consequently, we will cluster randomized GPs in the same region to promote local cooperation among professionals.

It has been reported that patient’s and healthcare provider’s expectations, based on previous experiences and representations, are associated with prognosis of patients with nonspecific LBP (50–52). Regarding patients, we believe that our randomized study design will limit the confusion bias from the heterogeneity in baseline patients’ expectations. Similarly, concerning healthcare providers, we expect that the randomized study design will equally distribute the healthcare providers’ expectations between the study groups at baseline. In addition, our study has been designed to evaluate whether the intervention will significantly change beliefs in healthcare provider’s, allocated to the Coordinated care group. This will be measured using the PABS (53). If required, an adjustment according to baseline levels has been planned.

In our study, the study intervention does not allow either the patients or providers to be completely blinded to the study group. Indeed, the study intervention involves not only specific actions from the GP but also informed patient participation, as an active partner in their care. We have done our best to limit information about the alternative group. For example, we created two different patients’ information and consent letters: one for each group. The information provided to patients has been adapted to the group allocated. In addition, the flow of information between groups are unlikely since GPs and their patients allocated the same cluster share the same geographical area, which differs to that of other clusters.

As in most clinical trials, our study may be affected by the “Hawthorne effect” or “trial effect” (54, 55). This effect concerns the changing of behavior due to study participation and the feeling of being observed. This may affect GPs in the control group, performing more careful treatment than usual, but may also occur in GPs allocated to the intervention group, with them having higher expectations and increased motivation. Therefore, it is difficult to estimate how, and to what extent, this effect could bias the study results.

This study will provide valuable data concerning the management of LBP in primary care in France. Overall, it will allow us to evaluate the benefit of a coordinated approach to LBP management, from the patients’ perspective, among other outcomes: to alleviate the associated disability, attenuate the pain, and to promote the return to work in patients suffering from subacute or recurrent acute LBP.

## Future directions and clinical implications

Our study design is pragmatic and based on current healthcare practices in French primary care. Consequently, our results will have a high potential for transferability. They may support early collaboration between GPs, physiotherapists, and OPs for treating patients with subacute or recurrent acute LBP in primary care. More largely, they may also advocate for reinforcing interdisciplinary collaborative practices around patients having musculoskeletal disorders, pain syndromes, or other types of chronic conditions.

## Trial status

On the 5th of May 2023, 19 clusters have been initiated with the approval to include patients: 41 patients have been enrolled.

## Ethics statement

The CO.LOMB study was conducted in accordance with the guidelines of the Declaration of Helsinki, and respecting French and European regulations, including the European General Data Protection Regulation and the requirements of the French “Commission Nationale de l’Informatique et des Libertés”. The study has been approved by an independent French ethics committee, the “CPP Ile de France XI” (Study number 20.01298.067400-MS02). All participants provided written informed consent before participating in the study.

## Author contributions

AR-R, CyB, EP-S, AP, MP, and CéB: study concept and design. AR-R, SC, TB, MG, MP, and CéB: acquisition of data. AR-R and MP: drafting of the manuscript. AR-R, EP-S, SC, TB, JV, and CéB: critical revision of the manuscript for important intellectual content. AR-R, CyB, SC, TB, EP-S, and CéB: obtaining funding. AL: administrative, technical, or material support. AR-R: supervision. CyB and JV: clinical training. All authors contributed to the article and approved the submitted version.

## Funding

This study is funded by a grant called PREPS (“Research Program on Performance of Healthcare Systems”) from the French Ministry of Health (PREPS-18-0407).

## Conflict of interest

The authors declare that the research was conducted in the absence of any commercial or financial relationships that could be construed as a potential conflict of interest.

## Publisher’s note

All claims expressed in this article are solely those of the authors and do not necessarily represent those of their affiliated organizations, or those of the publisher, the editors and the reviewers. Any product that may be evaluated in this article, or claim that may be made by its manufacturer, is not guaranteed or endorsed by the publisher.
